# Transcriptomic, proteomic, and physiological comparative analyses of flooding mitigation of the damage induced by low-temperature stress in direct seeded early *indica* rice at the seedling stage

**DOI:** 10.1186/s12864-021-07458-9

**Published:** 2021-03-12

**Authors:** Wenxia Wang, Jie Du, Liming Chen, Yongjun Zeng, Xueming Tan, Qinghua Shi, Xiaohua Pan, Ziming Wu, Yanhua Zeng

**Affiliations:** Key Laboratory of Crop Physiology, Ecology and Genetic Breeding, Ministry of Education / Collaborative Innovation Center for the Modernization Production of Double Cropping Rice / College of Agronomy, Jiangxi Agricultural University, Nanchang, 330045 China

**Keywords:** Rice, Low temperature, Flooding, Proteome, Transcriptome, Physiological traits

## Abstract

**Background:**

Low temperature (LT) often occurs at the seedling stage in the early rice-growing season, especially for direct seeded early-season *indica* rice, and using flooding irrigation can mitigate LT damage in rice seedlings. The molecular mechanism by which flooding mitigates the damage induced by LT stress has not been fully elucidated. Thus, LT stress at 8 °C, LT accompanied by flooding (LTF) and CK (control) treatments were established for 3 days to determine the transcriptomic, proteomic and physiological response in direct seeded rice seedlings at the seedling stage.

**Results:**

LT damaged chloroplasts, and thylakoid lamellae, and increased osmiophilic bodies and starch grains compared to CK, but LTF alleviated the damage to chloroplast structure caused by LT. The physiological characteristics of treated plants showed that compared with LT, LTF significantly increased the contents of rubisco, chlorophyll, PEPCK, ATP and GA_3_ but significantly decreased soluble protein, MDA and ABA contents. 4D-label-free quantitative proteomic profiling showed that photosynthesis-responsive proteins, such as phytochrome, as well as chlorophyll and the tricarboxylic acid cycle were significantly downregulated in LT/CK and LTF/CK comparison groups. However, compared with LT, phytochrome, chlorophyllide oxygenase activity and the glucan branching enzyme in LTF were significantly upregulated in rice leaves. Transcriptomic and proteomic studies identified 72,818 transcripts and 5639 proteins, and 4983 genes that were identified at both the transcriptome and proteome levels. Differentially expressed genes (DEGs) and differentially expressed proteins (DEPs) were significantly enriched in glycine, serine and threonine metabolism, biosynthesis of secondary metabolites, glycolysis/gluconeogenesis and metabolic pathways.

**Conclusion:**

Through transcriptomic, proteomic and physiological analyses, we determined that a variety of metabolic pathway changes were induced by LT and LTF. GO and KEGG enrichment analyses demonstrated that DEGs and DEPs were associated with photosynthesis pathways, antioxidant enzymes and energy metabolism pathway-related proteins. Our study provided new insights for efforts to reduce the damage to direct seeded rice caused by low-temperature stress and provided a breeding target for low temperature flooding-resistant cultivars. Further analysis of translational regulation and metabolites may help to elucidate the molecular mechanisms by which flooding mitigates low-temperature stress in direct seeded early *indica* rice at the seedling stage.

**Supplementary Information:**

The online version contains supplementary material available at 10.1186/s12864-021-07458-9.

## Background

Rice (*Oryza sativa* L.) is the staple food for more than half of the world’s population [1–3]. Due to the advances made since the Green Revolution of the 1960s, rice yield has increased considerably. As a typical thermophilic crop, the growth and development of rice are susceptible to changes in temperature, especially decreases in temperature [4, 5]. With the rise of global temperature and frequent occurrence of extreme weather, direct seeded rice is strongly affected by weather compared with traditional transplanting, especially rice seedlings emergence [6, 7]. After direct sowing, heavy rainfall and “cold spell in later spring” disaster make it easy for a large area of rotten seeds and rotten seedlings, resulting in uneven seedling emergence and poor population growth, ultimately reducing the production of direct seeded rice [8, 9]. In addition, due to global warming and changes in production habits, sowing dates have become earlier than before, which increases the probability that direct seeded rice will suffer from low temperature to some extent, especially in the seedling stage of direct seeded early rice, resulting in irreversible cold-tolerant growth of seedlings [10, 11]. In 2008 and 2010, due to the low temperature and severe cold stress in China, the emergence rate of direct seeded early rice decreased by 38–55%, and rice yield notably decreased [12]. At the same time, although rice is a water-loving crop, its growth and development are also affected by long-term flooding stress, resulting in anaerobic respiration of roots and leaves, which produces alcohol toxicity and reduces leaf photosynthesis [13]. Therefore, it is urgently important to perform in-depth research on the response mechanism to low-temperature stress of direct seeded early rice and prevention measures to alleviate this stress and improve the efficiency and stable yield of direct seeded rice.

In direct seeded rice production, when low temperatures occur, farmers often reduce the damage of low temperature to seedlings by irrigating a certain amount of shallow water to protect the seedlings [14]. Through this measure, the survival rate of seedling emergence can effectively increase, and the production risk of direct seeded rice can be reduced [15, 16]. To date, many studies have investigated rice cultivation, physiological traits, genetic mechanisms and other aspects of injuries induced by low-temperature stress [17–19] and flooding stress [20, 21]. The effects of flooding on the physiological and ecological characteristics of rice under low temperature have been reported in previous studies, but the conclusions reached by these studies were inconsistent [22, 23]. It has been reported that flooding at the seedling stage significantly increases plant height and internodes and accelerates the growth of germ sheaths, which can effectively reduce the dead seedling rate [24, 25]. The effect of different flooding depths on rice varies significantly. Moderate flooding can stimulate changes in physiological characteristics in plants, thereby promoting the growth of plant height and causing rice to exhibit better adaptability [26]. When the seedlings encounter low-temperature stress, moderate flooding could increase temperature for heat preservation effects, alleviate the accumulation of reactive oxygen species, intensify membrane lipid peroxidation under low temperature conditions, and slow the regulation of endogenous hormones in plants [27]. The direct damage to early rice seedlings caused by low temperature may thus be prevented [28]. At present, most studies on this topic have focused on agronomic traits or related physiological characteristics under different flooding layers [29, 30]. However, the molecular mechanism governing the mitigation effect of shallow flooding irrigation on low-temperature stress in direct seeded early *indica* rice seedlings has rarely been reported.

With the rapid development of biotechnology, an increasing number of studies on rice in response to different stresses have been analysed in depth by transcriptomic technologies [31, 32]. Transcriptome analysis is rapid and comprehensive and has been constructed and annotated to assist in the identification of differentially expressed genes (DEGs) in different plant populations [33]. However, the analysis of gene expression by measuring mRNA is limited, as mRNA is defined as indirect and temporary messages that transmit information. In contrast, proteins play a direct role in biological processes and are the basis of organisms [34]. Protein is the embodiment and executor of plant functions, which not only regulates plant stress tolerance by changing the catalytic activity of enzymes, but also acts as a transcription factor to regulate the expression of other genes [35–37]. Through the combination of the transcriptome and proteome, many differentially expressed proteins (DEPs) have been identified, and metabolic pathways have been found [38]. On this basis, many DEGs related to metabolic pathways have also been identified, providing a molecular mechanism for detecting responses to environmental stress.

At present, many studies have elucidated the mechanisms by which low-temperature stress or flooding stress affect rice seedlings from the aspects of proteomics and transcriptomics [39, 40]. However, the changes in transcriptomics and proteomics associated with low-temperature flooding have not been elucidated. The molecular mechanism of the mitigating effect, rather than superposition effect, of the hypoxic treatment caused by flooding under low temperature is a scientific problem that merits further study. This study combined transcriptomics and 4D-label-free quantitative proteomic analysis to explore the molecular mechanism by which flooding mitigates low-temperature stress on direct seeded early *indica* rice at the seedling stage. In this study, we identified genes and proteins that were obtained from Illumina-Hiseq and 4D-label-free searching for likely protein identification in Gene Ontology (GO), Kyoto Encyclopedia of Genes and Genomes (KEGG), Klustersof eukaryotic Orthologous Groups (KOG), Swissport and UniProt databases, respectively, and focused on the DEGs and DEPs involved in the flooding-mediated mitigation of low-temperature stress. The results of this study may help to guide the breeding and cultivation of low temperature-tolerant crop cultivars. This research also provides evidence for meteorological disaster mitigation and low temperature-induced damage prevention.

## Results

### Transmission electron microscopic observation of chloroplast structure

**Fig. 1:**
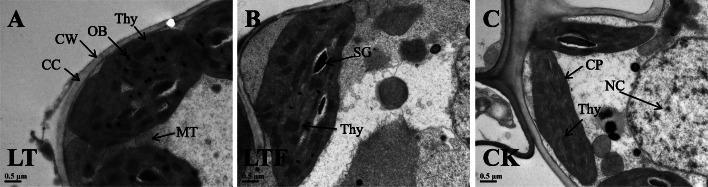
Transmission electron microscope analysis of the top leaves at rice seedlings stage after low temperature and low temperature flooding stress. LT: low temperature, LTF: low temperature flooding, CK: control. Thy: thylakoid lamellae, OB: osmiophilic body, CP: chloroplast, CW: cell wall, MT: mitochondrion, SG: starch grain, NC: nucleus

In this study, transmission electron microscopy was employed to compare the differences in chloroplasts structural of early *indica* rice seedlings after 3 days between LT and LTF groups. The results showed that chloroplasts were regular boat-shaped or spindle-shaped and that thylakoid lamellae were clearly arranged close to the inner wall of cells in CK (Fig. 1c). Compared with CK, chloroplasts began to degrade, exhibiting distorted and loosely structured shapes in LTF (Fig. 1b); however, in LT, chloroplasts were severely degraded, thylakoid lamellae were seriously damaged, and osmiophilic bodies and starch grains increased gradually (Fig. 1a). The damage to chloroplast structure in LTF was less than that observed in LT. These results showed that chloroplasts were affected to some extent by low-temperature stress, and flooding could alleviate low-temperature damage to the chloroplast structure.

### Analysis of photosynthesis activity and endogenous hormone content

**Fig. 2:**
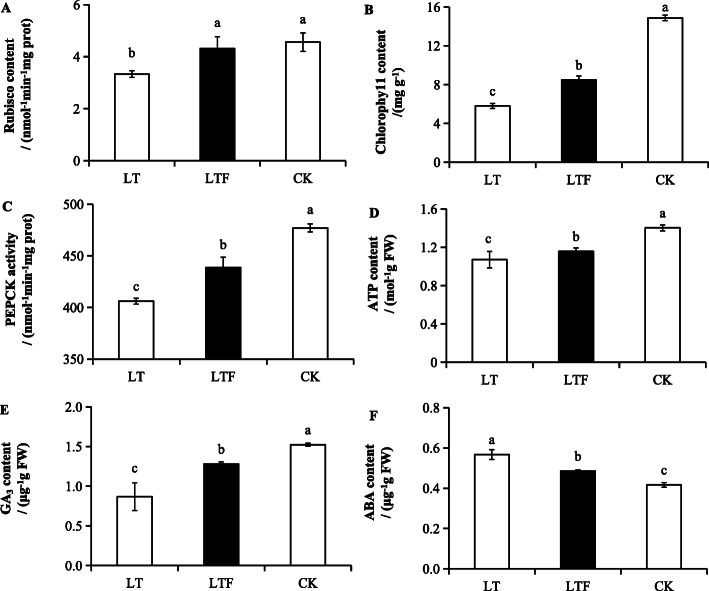
Analysis photosynthate activity and endogenous hormone content. **a** rubisco content, **b** chlorophyll content, **c** PEPCK activity, **d** ATP content, **e** GA_3_ content. **f** ABA content. Error bars represent standard deviation (*n* = 3). Data are mean ± SD. The data were detected by Tukey’s honest significant difference (HSD), and different lowercase letters indicated significant differences at *P* < 0.05. LT: low temperature, LTF: low temperature flooding, CK: control

This study showed that the rubisco content of LT was significantly decreased by 26.97% (*P* < 0.05) compared to that of CK, but there was no significant difference between LTF and CK (Fig. 2a). The PEPCK activity, chlorophyll content and ATP content of LT and LTF were significantly decreased (*P* < 0.05) compared with CK, and those indexes were lower in LT than in LTF (Fig. 2b, c, d). Compared with CK, the GA_3_ content of pro-growth hormones in LT and LTF decreased significantly (*P* < 0.05) (Fig. 2e). In contrast, the ABA content of anti-growth hormones in LT and LTF increased significantly by 35.71 and 16.67% (*P* < 0.05), respectively (Fig. 2f). There were similar trends in GA_3_ and ABA between LT and LTF, which reached a significant level. These results indicated that flooding could improve photosynthetic activity and endogenous hormone content under low-temperature stress in direct seeded early *indica* rice at the seedling stage.

### Analysis of soluble protein content, antioxidase and osmotic regulatory substances

**Fig. 3:**
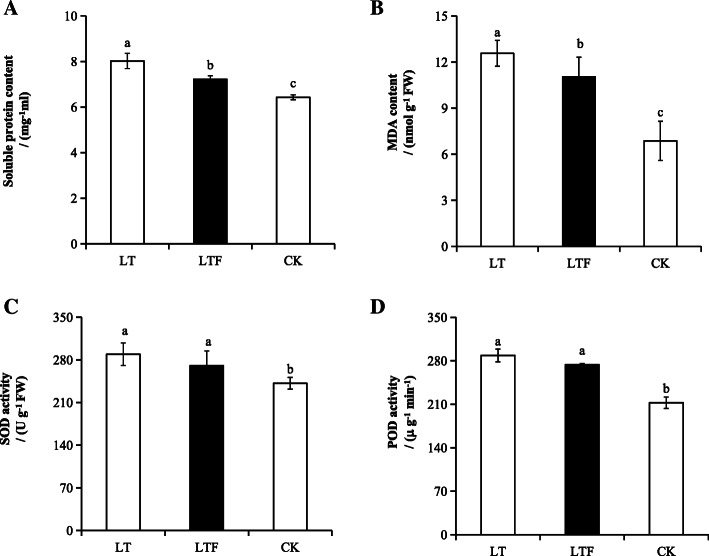
Analysis of soluble protein content, antioxidase and osmotic regulatory substances. **a** soluble protein content, **b** MDA content, **c** SOD activity, **d** POD activity. Error bars represent standard deviation (*n* = 3). Data are mean ± SD. The data were detected by Tukey’s honest significant difference (HSD), and different lowercase letters indicated significant differences at *P* < 0.05. LT: low temperature, LTF: low temperature flooding, CK: control

This study showed that the contents of soluble protein and MDA in LT and LTF increased significantly compared with CK, and LT also significantly increased soluble protein and MDA compared to LTF (*P* < 0.05) (Fig. 3a, b). In addition, LT and LTF significantly increased the activities of SOD and POD (*P* < 0.05) compared with CK, and there were no significant differences between LT and LTF, although SOD and POD were higher in LT than in LTF (Fig. 3c, d).

### Identification of DEPs and DEGs

**Fig. 4:**
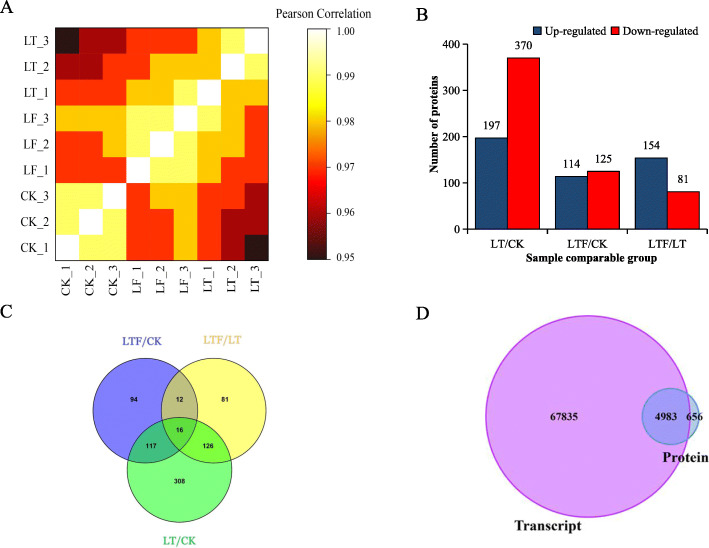
**a** Pearson correlation coefficient thermograph of protein quantification. A Pearson coefficient closer to − 1 indicates a negative correlation, a coefficient closer to 1 indicates a positive correlation and a coefficient closer to 0 indicates no correlation. **b** Summary of up-regulated and down-regulated of DEPs between the treatment groups (LT, LTF) and control group (CK). **c** Venn diagram the DEPs between the treatment groups (LT, LTF) and control group (CK). **d** Comparison of transcriptome and proteome identification. LT: low temperature, LTF: low temperature flooding, CK: control

To evaluate the reliability of the data through proteomic analysis, the Pearson correlation coefficient was calculated for each of three samples, which indicated good reproducibility of the three biological replicates in each treatment (Fig. 4a). In addition, a total of 412,489 spectra were detected, 236,880 of which could be matched to peptides in the database, and 28,934 of the peptides were unique. In total, 5639 proteins could be identified, and 4518 proteins were experimentally quantified (Table 1).

**Table 1:** MS/MS spectrum database search analysis summary

Total spectrums	Matched spectrums	Peptides	Unique peptides	Identified proteins	Quantifiable proteins
412,489	236,880	31,364	28,934	5639	4518

Proteins with fold change (FC) values > 1.5 or (FC) values < 0.67 (*P* < 0.05) between the treatment (LT, LTF) and control groups (CK) were regarded as DEPs, and DEPs were hence considered low temperature- and low temperature flooding-responsive proteins at the seedling stage. There were 567 DEPs between LT and CK, 239 DEPs between LTF and CK, and 235 DEPs between LTF and LT. The number of upregulated and downregulated DEPs is shown in Fig. 4b, and the three groups had 16 DEPs in common (Fig. 4c).

**Fig. 5:**
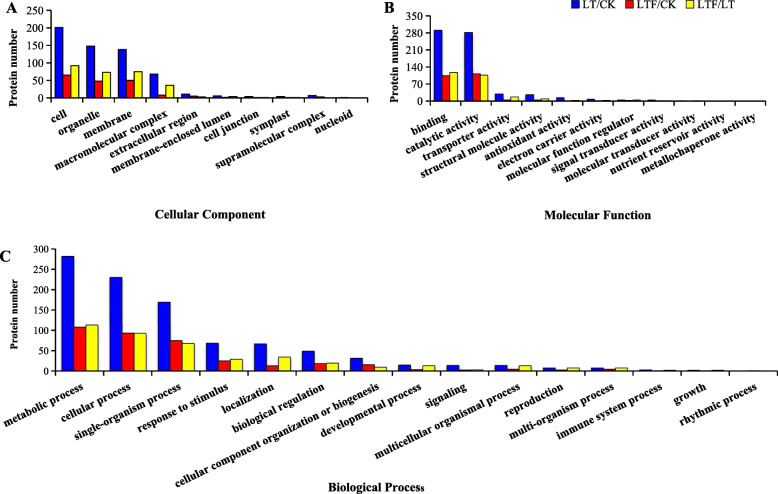
The transcript and its corresponding protein expression scatter diagram. LT: low temperature, LTF: low temperature flooding, CK: control

In this study, 72,818 transcripts and 5639 proteins were identified by quantitative transcriptome and proteome studies. A total of 4983 genes were identified at both the transcriptome and proteome levels (Fig. 4d). The correlation coefficient between transcripts and proteins in the LT and CK treatment groups was 0.19, that in the LTF and CK treatment groups was 0.25 and that in the LT and LTF treatment groups was 0.22. This finding indicates that the correlation degree of samples in each treatment group is low, and these results are largely consistent with the expected results (Fig. 5).

### Gene functional description and GO analysis

To annotate the function of low-temperature flooding-responsive proteins, the protein IDs were searched in the NCBI database (https://www.ncbi.nlm.nih.gov/) and/or the UniProt database (http://www.uniprot.org/). For the DEPs between LT and CK, 197 upregulated proteins and 369 downregulated proteins exhibited annotated functions, and 1 downregulated protein remained uncharacterized (Additional file 1: Dataset S1). For the DEPs between LTF and CK, both 114 upregulated proteins and 125 downregulated proteins could be annotated with functions (Additional file 2: Dataset S2). For the DEPs between LTF and LT, both 154 upregulated proteins and 81 downregulated proteins showed annotated functions (Additional file 3: Dataset S3).

**Fig. 6:**
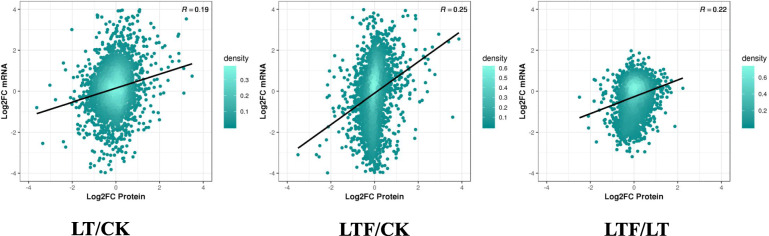
GO classification analysis of DEPs between LT, LTF and CK. **a** cellular component, **b** molecular function, **c** biological process. LT: low temperature, LTF: low temperature flooding, CK: control

To determine the cellular component (CC), molecular function (MF) and biological process (BP) categories of GO for the low temperature- and low temperature flooding- responsive proteins, we searched their protein IDs from the GO database. GO analysis showed that the DEPs were involved in 14 subgroups of BP (Fig. 6a), ten subgroups of CC (Fig. 6b), and ten subgroups of MF (Fig. 6c) between LT and CK. The main biological process categories were metabolic process (30%), cellular process (25%), single-organism process (18%), response to stimulus (7%), localization (7%), biological regulation (5%) and cellular component organization or biogenesis (4%). The cellular component categories were cell (34%), organelle (25%), membrane (24%), and macromolecular complex (12%). The molecular function categories were binding (44%), catalytic activity (42%), transporter activity (5%), structural molecule activity (4%), and antioxidant activity (2%) (Additional file 4: Fig. S1).

GO analysis showed that the DEPs were associated with 13 subgroups of BP (Fig. 6a), nine subgroups of CC (Fig. 6b), and ten subgroups of MF (Fig. 6c) between LTF and CK. The biological process categories were metabolic process (30%), cellular process (26%), single-organism process (21%), and response to stimulus (7%), biological regulation (5%), cellular component organization or biogenesis (4%), and localization (4%). The cellular component categories were cell (36%), membrane (28%), organelle (26%), macromolecular complex (4%), and extracellular region (3%). The molecular function categories were catalytic activity (47%), binding (44%), structural molecule activity (2%), and transporter activity (2%) (Additional file 5: Fig. S2).

GO analysis showed that the DEPs were involved in 14 subgroups of BP (Fig. 6a), eight subgroups of CC (Fig. 6b), and nine subgroups of MF (Fig. 6c) between LTF and LT. The biological process categories were metabolic process (27%), cellular process (19%), single-organism process (17%), localization (10%), response to stimulus (7%), biological regulation (5%), developmental process (3%), multicellular organismal process (3%), cellular component organization or biogenesis (3%), and reproduction (3%). The cellular component categories were membrane (33%), cell (29%), organelle (23%), and macromolecular complex (12%). The molecular function categories were binding (45%), catalytic activity (41%), transporter activity (7%), and structural molecule activity (3%) (Additional file 6: Fig. S3).

### Protein–protein interaction

The functional DEPs of all annotations were utilized to analyse protein interactions. This analysis demonstrated that most enzymatic proteins and proteins related to biosynthesis of secondary metabolites, monobactam biosynthesis, metabolic pathways, pentose phosphate pathway, fructose and mannose metabolism, glycolysis/gluconeogenesis, glycine, serine and threonine metabolism, arachidonic acid metabolism, biosynthesis of amino acids, phenylalanine, tyrosine and tryptophan biosynthesis and proteasome-related protein interactions were affected by LT and CK (Additional file 7: Fig. S4). Most enzymatic proteins and metabolic pathways, biosynthesis of secondary metabolites, carotenoid biosynthesis, ribosome biogenesis in eukaryotes, glycolysis/gluconeogenesis, glycine, serine and threonine metabolism photosynthesis and thiamine metabolism were observed for the interaction between LTF and CK (Additional file 8: Fig. S5). Most enzymatic proteins and photosynthesis-antenna proteins and photosynthesis-related protein interactions were affected by LTF and LT (Additional file 9: Fig. S6). In the LT/CK and LTF/CK comparison groups, the relevant DEPs in the metabolic pathway included A2X8P7, A2XLW5, A2XYG6 and B8AYU2, which indicated energy metabolism-related proteins. In the glycine, serine and threonine metabolic pathways, the relevant DEPs had A2YMZ1 and A2YCB9, which indicated photosynthesis-related proteins. In this study, the photosynthesis pathway and energy metabolism pathway were highly enriched under low temperature and low temperature flooding. This result showed that low temperature flooding played an important role in regulating the photosynthetic capacity of rice leaves. Consistent with our GO analysis findings, the majority of proteins were determined to be involved in photosynthesis and metabolic processes. We focused on proteins related to photosynthesis and metabolism at the proteomic level.

### KEGG pathway analysis

All of the DEGs and DEPs were analysed for the KEGG over-representation of pathways to obtain functional insights into the differences between LT, LTF and CK treatments. The significantly (*P* < 0.01) enriched KEGG pathways are shown in Table 2. The KEGG pathways (ordered by rank) were monobactam biosynthesis, glycine, serine and threonine metabolism, biosynthesis of secondary metabolites, pentose phosphate pathway, biosynthesis of amino acids, metabolic pathways, arachidonic acid metabolism, glycolysis/gluconeogenesis, proteasome, phenylalanine, tyrosine and tryptophan biosynthesis, and fructose and mannose metabolism between LT and CK. The KEGG pathways (ordered by rank) were thiamine metabolism, ribosome biogenesis in eukaryotes, carotenoid biosynthesis, biosynthesis of secondary metabolites, metabolic pathways, glycine, serine and threonine metabolism, and glycolysis/gluconeogenesis between LTF and CK. The KEGG pathways (ordered by rank) were photosynthesis and photosynthesis-antenna proteins between LTF and LT. In the LT/CK and LTF/CK comparison groups, the co-enriched KEGG pathways were glycine, serine and threonine metabolism, biosynthesis of secondary metabolites, metabolic pathways, and glycolysis/gluconeogenesis.

**Table 2:** Enriched KEGG Pathways associated with DEGs and DEPs

Pathway name	Pathway ID	Number of molecules		*P* value
		Mapping	All	
KEGG Pathways between LT and CK				
Monobactam biosynthesis	osa00261	2	13	5.44E-04
Glycine, serine and threonine metabolism	osa00260	4	26	4.69E-04
Biosynthesis of secondary metabolites	osa01110	10	13	1.16E-04
Pentose phosphate pathway	osa00030	2	13	8.82E-03
Biosynthesis of amino acids	osa01230	5	26	8.08E-03
Metabolic pathways	osa01100	11	13	2.71E-03
Arachidonic acid metabolism	osa00590	2	26	1.96E-03
Glycolysis / Gluconeogenesis	osa00010	2	13	4.85E-02
Proteasome	osa03050	2	26	4.00E-02
Phenylalanine, tyrosine and tryptophan biosynthesis	osa00400	2	26	2.51E-02
Fructose and mannose metabolism	osa00051	2	13	1.44E-02
KEGG Pathways between LTF and CK				
Thiamine metabolism	osa00730	2	14	9.73E-04
Ribosome biogenesis in eukaryotes	osa03008	3	20	5.79E-03
Carotenoid biosynthesis	osa00906	2	14	4.45E-03
Biosynthesis of secondary metabolites	osa01110	9	14	2.01E-03
Metabolic pathways	osa01100	12	14	1.35E-03
Glycine, serine and threonine metabolism	osa00260	2	20	3.28E-02
Glycolysis / Gluconeogenesis	osa00010	3	20	1.67E-02
KEGG Pathways between LTF and LT				
Photosynthesis - antenna proteins	osa00196	2	9	2.53E-04
Photosynthesis	osa00195	3	9	2.12E-04

The pathway ranking in this table is in order from high to low between LT, LTF and CK. The “mapping” number represents the number of annotated DEGs and DEPs in the pathway, while the “all” number represents the total number of proteins in the pathway

*LT* low temperature, *LTF* low temperature flooding, *CK* control

### Analysis of DEGs by qRT-PCR

**Fig. 7:**
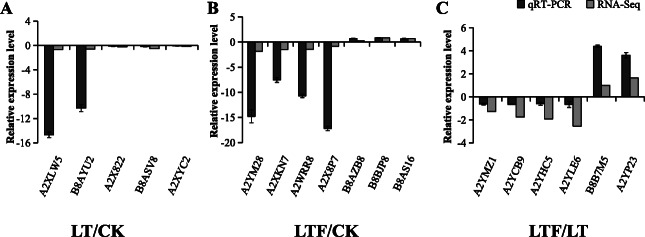
Comparison of the expressions of RNA-Seq and qRT-PCR results. **a**: LT/CK, **b**: LTF/CK, **c**: LTF/LT. The transcript levels of the selected genes were each normalized to that of the 18 s gene. The relative expression of qRT-PCR transcript was calculated according to the standard curve and normalized to 18 s gene. The calculated data (mean ± SD) of three individual (*n* = 3). LT: low temperature, LTF: low temperature flooding, CK: control

To verify the transcriptome results, 18 related genes, including five upregulated genes and 13 downregulated genes, were analysed. The mRNA expression of A2XLW5, B8AYU2, A2X822, B8ASV8 and A2XYC2 was downregulated between LT and CK (Fig. 7a). The mRNA expression levels of A2YM28, A2XKN7, A2WRR8 and A2X8P7 were downregulated between LTF and CK, and B8AZB8, B8BJP8, and B8AS16 were significantly upregulated between LTF and CK (Fig. 7b). In LTF and LT, A2YMZ1, A2YCB9, A2YHC5 and A2YLE6 were determined to be downregulated, and B8B7M5 and A2YP23 were observed to be upregulated (Fig. 7c). These results confirmed that the transcriptome results reflected the relative expression of each gene, in which upregulated or downregulated genes in qRT-PCR were entirely consistent with transcriptome trends. Therefore, the transcriptome results were reliable.

## Discussion

### Effect of flooding on photosynthesis under low-temperature stress

The effects of LT stress on plants are multifaceted, such as growth retardation, physiological metabolic disorders, inhibition of photosynthetic efficiency, and cell membrane damage [41]. Photosynthesis is the most sensitive process for plants in response to LT stress, and chlorophyll is the catalyst for photosynthesis in plants [42, 43]. Our study showed that LT significantly decreased the chlorophyll content, and degraded chloroplasts and membrane lipids, resulting in numerous and large osmiophilic granules, since the expression of chlorophyll-binding proteins (A2YCB9, A2XAH0, A2XN43) induced by LT was significantly downregulated compared with CK (Additional file 1: Dataset S1). LT-induced chlorophyll-associated protein degradation may lead to inhibited photosynthesis that is directly linked with photosynthesis metabolism. This phenomenon could conceivably represent one of the main causes of rice susceptibility to LT stress [44], and our study obtained similar findings (Additional file 3: Dataset S3). Additionally, previous studies found that submergence caused O_2_^−^ and CO_2_^−^ deficiency by limiting ambient gas exchange, reduced the light available for photosynthesis and disrupted cellular energy production and ionic homeostasis [45]. However, this study indicated that 5–6 cm flooding had a high chlorophyll content under low-temperature stress, which was attributed to the high expression levels of chlorophyll-related proteins (A2XCH9 and B8B1R3) induced by LTF. In field production, the minimum water temperature is often 3 °C–4 °C higher than the minimum air temperature; therefore, moderate flooding could increase water temperature and alleviate the direct damage to early rice seedlings caused by low temperature, thereby reducing losses in rice production [26, 46].

Chlorophyll is also the main pigment of plant photosynthesis, and its biosynthesis is a series of enzymatic reactions [47]. Low-temperature stress might reduce the activities of key enzymes related to chlorophyll synthesis and affect the process of chlorophyll synthesis, while the effect of flooding on chlorophyll synthesis enzymes is influenced by the duration and depth of flooding [48, 49]. Rubisco and ATP synthases are key enzymes that catalyse the photosynthetic carbon cycle and are highly sensitive to the external environment [50, 51]. ATP synthase β subunit content was significantly reduced at 6 °C in tomato [52], rubisco activity is one of the major limiting factors of photosynthesis, and the photosynthetic capacity of leaves can be enhanced by increasing the rubisco content under low-temperature conditions [53, 54]. Our study had similar results on ATP content, and rubisco and PEPCK activities in rice seedlings, due to the lower expression of ATP synthase-associated proteins (A2XF96 and B8AC70), rubisco-associated protein (A2Z7X6) and pyruvate kinase-associated proteins (A2XEP1 and B8AYC1) (Additional file 1: Dataset S1). However, moderate flooding could promote the growth and development of rice seedlings under low-temperature stress and mitigate the damage caused by low temperature [55, 56]. The present results also demonstrated that LTF significantly increased ATP content, rubisco and PEPCK activities that were associated with high ATP-associated proteins (A2XF96 and B8AMY3), rubisco-associated protein (B8BIM0) and PEPCK-associated proteins expression (B8ACJ0 and B8ACJ0) (Additional file 3: Dataset S3). Flooding could maintain the preferable sheath growth point and root system under low temperature conditions, which improved the ambient temperature tolerance to low temperature of rice, and the increase in rubisco and PEPCK activities significantly improved photosynthetic efficiency within a certain range.

### Effect of flooding on the antioxidant system under low-temperature stress

When rice was subjected to LT stress, the balance of intracellular oxygen metabolism was disturbed, resulting in the production of reactive oxygen species (ROS). The accumulation of ROS might cause oxidative stress, but rice can regulate it through antioxidant enzymes (such as POD and SOD) to resist the toxic effects of reactive oxygen free radicals under LT stress [57–59]. The accumulation of ROS scavenging enzymes has been determined to be one of the most important responses during LT stress [60]. Moderate flooding could further enhance the antioxidant capacity of plants, inhibit membrane peroxidation and reduce the aggravation of leaf peroxidation damage [61]. Our study found that under LT treatment, the key proteins of the antioxidant pathway, including ROS scavenging (A2XX54 and B8AKJ8), oxidoreductase (A2YAP5) and glutathione peroxidase (A2X822 and B8ASV8), were significantly upregulated (Additional file 1: Dataset S1). Meanwhile, superoxide dismutase activity (A2XAA0), oxidoreductase (A2YAP5) and glutathione peroxidase (B8ASV8) were significantly upregulated between LTF and CK (Additional file 2: Dataset S2), which was consistent with the increase in the activities of antioxidant enzymes, such as POD and SOD, in LT and LTF. The results of this study were in keeping with the findings of a previous study [45]. LT stress upregulated ROS scavenging, redox adjustment, cryoprotection and defence detoxification-related proteins. To survive under LT stress, plants induce the antioxidant enzyme system to remove reactive oxygen groups to prevent these groups from destroying the cell and to enhance their stress tolerance [62–64]. However, because the temperature was too low and the damage to rice seedlings was serious, excessive ROS attacked rice, resulting in cell structure damage and metabolic disorder, which might be one of the crucial reasons for the damage to chloroplast structure. In this study, the expression of peroxidase protein (B8ARU3) was significantly downregulated in the LTF and LT groups compared to the LTF group. Flooding plays a role in protecting seedlings from the direct stress of LT because shallow flooding could improve the antioxidant enzyme protection system of direct seeded rice seedlings under LT. The cold resistance of rice may be the result of the coordinated action of antioxidant enzymes and various resistance factors [65].

MDA is the final product of membrane lipid peroxidation, and the change in its content is one of the important indicators of the degree of plasma membrane damage [66]. Our study found that LTF significantly reduced MDA content, as the expression of lipid-associated proteins (A2ZFY8 and A2X7M2) was significantly lower than that of LT (Additional file 3: Dataset S3). This finding indicated that LT induced disintegration of leaf cell membranes and electrolyte leakage in the plants, whereas LTF could contribute to mitigating the involvement of lipoxygenases in membrane lipid peroxidation and reducing damage to the cell membrane system.

### Effect of flooding on metabolic regulation under low-temperature stress

Abscisic acid, as an endogenous hormone, is widely found in plants and plays an important role in enhancing plant stress tolerance [67]. The results indicated that the ABA content and the expression of ABA-binding protein (B8APF3) were significantly upregulated in the comparison of the LT and CK groups (Additional file 1: Dataset S1). Ma et al. [68] reported that overexpression of basic leucine zipper could promote the synthesis of ABA and improve the resistance of maize to low-temperature stress, and the results of our study were consistent with this finding. When the receptor cells of leaves and roots sense low-temperature stress, they induce gene expression and protein synthesis related to ABA precursor substances in plants, resulting in a large amount of ABA synthesis. ABA induces the expression of cold-resistance genes and promotes the synthesis of cold-resistance-specific proteins, thereby enhancing plant resistance to low-temperature stress. The dynamic balance regulation mechanism of different ABA contents in plants during growth and development can respond to different degrees of stress [69]. In contrast, the ABA content and ABA-associated protein expression (A2ZKG0) in LTF were significantly lower than those in LT (Additional file 3: Dataset S3). It is possible that flooding has a warming effect on the roots and stems of the aboveground parts. Agural et al. [70] reported that plant hormones, such as growth hormone and cytokinin, also affect plant growth and development during low-temperature stress. Our study showed that the GA_3_ content and GA_3_ synthesis-related proteins (A2XJL0 and B8BCQ6) in LT were significantly lower than those in CK (Additional file 1: Dataset S1). Low-temperature stress limited the activities of key enzymes in the GA_3_ signal transduction pathway and GA_3_ synthesis process, which decreased the content of GA_3_ and inhibited the normal growth and development of plants [71]. GA_3_ is a hormone that efficiently promotes cell division and elongation of roots and leaves, and plays an important role in the entire life cycle of plants [72]. In this study, the GA_3_ content and cell elongation protein expression (A2Z6Y9) were significantly upregulated in the LTF and LT groups (Additional file 3: Dataset S3). This finding may be observed because flooding promoted the transmission of GA_3_ signaling molecules in the signaling pathways under low-temperature stress and regulated the expression of GA_3_ biosynthesis-related genes and the synthesis of specific proteins, thereby facilitating the synthesis of a large number of GA_3_. In addition, soluble protein is an important osmotic regulator substance [73, 74]. In this study, LTF significantly decreased the soluble protein content compared to LT. This finding might be attributable to the decrease in the low-temperature stress effect after flooding and the response of plant osmoregulatory mechanisms to changes in environmental factors; thus, producing and accumulating less soluble proteins could ensure normal plant growth and development.

## Conclusions

In this study, transcriptomic, proteomic and physiological analyses were employed to investigate the molecular mechanism by which flooding mitigates low-temperature stress. The damage that low-temperature stress caused to chloroplast structures in rice leaves was alleviated by flooding. LTF significantly increased the contents of rubisco, chlorophyll, PEPCK, ATP and GA_3_ and enhanced photosynthesis and energy metabolism compared with LT. GO and KEGG enrichment analysis demonstrated that the DEGs and DEPs were significantly associated with the photosynthesis pathway, antioxidant enzymes and metabolic regulation. Our results may help to elucidate the physiological characteristics, and gene and proteins expression changes by which flooding mitigates low-temperature stress.

## Methods

### Plant materials and growth conditions

Based on previous experiments [75], the early *indica* rice cultivar Zhongjiazao 17 (ZJZ17) was selected for pot experiments, which is mainly generalized for rice production in Jiangxi Province. Zhongjiazao 17 bred by the China National Rice Research Institute (CNRRI) is inbred *indica* rice. The pot experiment was conducted at the Key Laboratory of Crop Physiology of Jiangxi Agricultural University (JXAU) of Nanchang City, Jiangxi Province, China (longitude: 115° 50′ E; latitude: 28° 46′ N). Rice was planted in plastic pots measuring 15.0 cm in height, 25.0 cm in length and 23.0 cm in width. Soil samples of the plastic pots were derived from the upper soil layer (0–20 cm) of the rice experiment paddy field at the experimental site located at the science and technology park of JXAU. The soil fertility properties were as the following: soil organic matter 30.35 g·kg^− 1^, total nitrogen (N) 2.4 g·kg^− 1^, available phosphorus (P) 25.17 mg·kg^− 1^, available potassium (K) 84.02 mg·kg^− 1^, and pH 6.1. The soil was naturally blown-dried, and crushed by a soil grinder (FT-1000A, Changzhou WIK Instrument Manufacturing Co. Ltd., China), and then sifted by a 100-mm mesh. Each pot filled approximately six kilograms of dry soil that was soaked in water for 2 weeks before direct seeding. Three grams compound fertilizer (N-P-K = 15-15-15%) was applied as base fertilizer 1 day before direct seeding. Other management measures were in accordance with local recommendations.

### Experimental design

Germinated seeds were selected for direct seeding, and all pots were subsequently placed in an artificial climate chamber with a diurnal temperature of 27/23 °C (day/night). After 20 days of direct seeding, one-third pots of rice seedlings with three leaves continued to be kept in a suitable temperature artificial climate chamber 27/23 °C (day/night) as a control (CK), and the two-thirds pots of rice seedlings were equally moved to another artificial climate chamber for low temperature (LT) and low temperature flooding (LTF) treatment. For low temperature (LT), the diurnal temperature was set to 10/6 °C (day/night), the period of low temperature treatment was 3 days, the light intensity was 100 μmol·m^− 2^ s^− 1^, the relative humidity (RH) of the artificial climate chamber was 75%, and potting soil remained moist. During the treatment, the positions of each pot were rotated from time to time to avoid the influence of light on seedling growth. For the low-temperature flooding treatment (LTF), the temperature and treatment period were both the same as those in the LT treatment, and the rice plants were maintained in a 5–6 cm flood water layer. For the control (CK), rice seedlings were grown in an artificial climate chamber in which the diurnal temperature was 27/23 °C (day/night), which kept the soil moist. Each treatment consisted of three replicates, and 20 pots constituted each replication. Under LT, LTF, and CK conditions, the relevant agronomic data were shown in Table S1 (Additional file 10).

### Transmission electron microscopic observation

After 3 days of low temperature and low temperature flooding, the middle and upper parts of the leaves were cut into 1 × 1-cm pieces with a blade and immediately placed into the 2.5% glutaric dialdehyde fixative, vacuumized until the sample was completely submerged, and fixed overnight at 4 °C; approximately 10 pieces were taken for each sample. The sample- processing methods were undertaken according to the method described by Huang [76].

### Physiological and biochemical characteristic

Enzymatic activity and endogenous hormone contents were measured on the third day of low-temperature and low-temperature flooding treatment. In each replicate experiment, three pots of plants were selected for each treatment group, and a 3-g leaf sample was collected and immediately frozen in liquid N_2_ and stored at − 80 °C until extraction. PEPCK activity and rubisco, chlorophyll, ATP, ABA, and GA_3_ contents [77–81] were measured using assay kits according to the manufacturer’s instructions (Cominbio, Suzhou, China). Soluble protein content, peroxidase (POD) activity, superoxide dismutase (SOD) activity and malondialdehyde (MDA) content were determined by configuration of solution [82–84].

### RNA isolation, library preparation, and sequencing

According to the RNA extraction scheme, the total RNA was separated using TRIzol reagent (Invitrogen, Waltham, MA, USA). RNA purity was checked by a Nano Photometer® spectrophotometer (IMPLEN, CA, USA). The integrity of the RNA was assessed using the RNA 6000 Nano Kit of the Bioanalyzer 2100 system (Agilent Technologies, CA, USA). The quality and quantity of the RNA samples were determined by a Nanodrop 2000 spectrophotometer (Thermo, USA).

The total amount of 3 μg RNA per sample was used as the input material for RNA sample preparation. Sequencing libraries were generated using the NEBNext® Ultra™ RNA Library Prep Kit for Illumina® (NEB, USA) following the manufacturer’s recommendations and index codes were added to attribute sequences to each sample. In brief, mRNA was purified from total RNA using poly-Toligo-attached magnetic beads. Fragmentation was carried out using divalent cations under elevated temperature in NEBNext First Strand Synthesis Reaction Buffer (5X). First strand cDNA was synthesized using random hexamer primers and M-MuLV Reverse Transcriptase (RNase H-). Second strand cDNA synthesis was subsequently performed using DNA polymerase I and RNase H. The remaining overhangs were converted into blunt ends via exonuclease/polymerase activities. After adenylation of the 3′ ends of DNA fragments, NEBNext adaptors with hairpin loop structures were ligated to prepare for hybridization. To preferentially select cDNA fragments 150 ~ 200 bp in length, the library fragments were purified with the AMPure XP system (Beckman Coulter, Beverly, USA). Then, 3 μL USER Enzyme (NEB, USA) was used with size- selected, adaptor-ligated cDNA at 37 °C for 15 min followed by 5 min at 95 °C before PCR. Then PCR was performed with Phusion High-Fidelity DNA polymerase, universal PCR primers and Index (X) Primer. Finally, PCR products were purified (AMPure XP system), and library quality was assessed with the Agilent Bioanalyzer 2100 system. After that step, the library preparation was sequenced on the Illumina HiSeq 2500/X platform, and 125/150-bp paired-end reads were generated. RSEM (version 1.2.15) was used to quantify the mRNA abundance.

### Differential expression analysis for RNA-seq data

Expected number of fragments per kilobase of transcript per million fragments mapped (FPKM) values were obtained using Cufflink software (version 2.1.1), which were used as values for normalized gene expression. Differential expression analyses of two comparison groups were performed using the DESeq R package (1.10.1). DESeq provides statistical routines for determining differential expression in digital gene expression data using a model based on the beta negative binomial distribution. The resulting *P*-values were corrected by the Benjamini-Hochberg method. Genes with an adjusted *P*-value < 0.01 and fold change > 2 found by DESeq were considered to be differentially expressed.

### Protein extraction, trypsin digestion and TMT labelling

The sample was first ground in liquid nitrogen, and then the powder was transferred to a 5-mL centrifuge tube. After that, four volumes of lysis buffer (8 M urea, 1% Protease Inhibitor Cocktail) were added to the cell powder, followed by sonication three times on ice using a high-intensity ultrasonic processor (Scientz, China). The samples were centrifuged at 12,000×g at 4 °C for 10 min and the debris was removed. Finally, the protein concentration was determined with a BCA kit (Beyotime, China) according to the manufacturer’s instructions.

For digestion, the protein solution was reduced with 5 mM dithiothreitol for 30 min at 56 °C and alkylated with 11 mM iodoacetamide for 15 min at room temperature in darkness. The protein sample was then diluted by adding 100 mM TEAB to urea concentrations less than 2 M. Finally, trypsin was added at a 1:50 trypsin-to-protein mass ratio for the first digestion overnight and a 1:100 trypsin-to-protein mass ratio for a second 4 h-digestion.

After trypsin digestion, the peptide was desalted by a Strata X C18 SPE column (Phenomenex, USA) and vacuum-dried. The peptide was reconstituted in 0.5 M TEAB and processed according to the manufacturer’s protocol for the TMT kit. The peptide mixtures were then incubated for 2 h at room temperature and pooled, desalted and dried by vacuum centrifugation.

### LC-MS/MS analysis and database search

The tryptic peptides were dissolved in 0.1% formic acid (solvent A) and directly loaded onto a homemade reversed-phase analytical column (15 cm length, 75 μm i.d.). The gradient consisted of an increase from 6 to 23% solvent B (0.1% formic acid in 98% acetonitrile) over 26 min, 23–35% in 8 min and climbing to 80% in 3 min then holding at 80% for the last 3 min, all at a constant flow rate of 400 nL/min on an EASY-nLC 1000 UPLC system (Thermo, Waltham, USA).

The peptides were subjected to an NSI source followed by tandem mass spectrometry (MS/MS) in a Q ExactiveTM Plus (Thermo) coupled online to UPLC. The electrospray voltage applied was 2.0 kV. The m/z scan range was 350 to 1.800 for full scan, and intact peptides were detected in the Orbitrap at a resolution of 70,000. Peptides were then selected for MS/MS using the NCE setting of 28, and the fragments were detected in the Orbitrap at a resolution of 17,500. A data-dependent procedure was employed that alternated between one MS scan followed by 20 MS/MS scans with 15.0 s dynamic exclusion. Automatic gain control (AGC) was set at 5E4. Fixed first mass was set as 100 m/z.

The resulting MS/MS data were processed using the MaxQuant search engine (v.1.5.2.8). Tandem mass spectra were searched against the human UniProt database concatenated with the reverse decoy database. Trypsin/P was specified as a cleavage enzyme allowing up to 4 missing cleavages. The mass tolerance for precursor ions was set as 20 ppm in the first search and 5 ppm in the main search, and the mass tolerance for fragment ions was set as 0.02 Da. Carbamidomethyl on Cys was specified as a fixed modification and acetylation modification and oxidation on Met were specified as variable modifications. The FDR was adjusted to < 1%, and the minimum score for modified peptides was set as > 40.

### Confirmation using qRT-PCR

To verify the accuracy of transcriptome and proteome results, 18 DEGs involved in LT, LTF and CK responses were selected for verification using qRT-PCR. The RNA used for qRT-PCR was the same with those used to construct cDNA library. The primers were designed using Primer 5.0 (Additional file 11: Table S2) and synthesized by Xiamen Life-Int Technology Co., Ltd. and 18 s (GenBank Accession NO. AK059783) acted as the reference gene.

### Statistical analysis

The data were analysed by analysis of variance (SAS Institute Inc., Cary, NC), and the means of different treatments were examined by Tukey’s honest significant difference (HSD) test to compare the differences at the probability level of 0.05.

## Supplementary Information

**Additional file 1 : Dataset S1.** Summaries and expression patterns of DEPs between LT and CK.

**Additional file 2 : Dataset S2.** Summaries and expression patterns of DEPs between LTF and CK.

**Additional file 3 : Dataset S3.** Summaries and expression patterns of DEPs between LTF and LT.

**Additional file 4 : Figure S1.** Functional categories of the identified differentially expressed proteins (DEPs) between LT and CK.

**Additional file 5 : Figure S2.** Functional categories of the identified differentially expressed proteins (DEPs) between LTF and CK.

**Additional file 6 : Figure S3.** Functional categories of the identified differentially expressed proteins (DEPs) between LTF and LT.

**Additional file 7 : Figure S4.** DEPs protein–protein interaction analysis between LT and CK.

**Additional file 8 : Figure S5.** DEPs protein–protein interaction analysis between LTF and CK.

**Additional file 9 : Figure S6.** DEPs protein–protein interaction analysis between LTF and LT.

**Additional file 10 : Table S1.** Effects of low-temperature and low temperature flooding on agronomic characters.

**Additional file 11 : Table S2.** Primers used for qRT-PCR verification of differently expressed genes.

## Data Availability

The mass spectrometry proteomics data have been deposited to the ProteomeXchange Consortium (http://proteomecentral.proteomexchange.org) via the PRIDE [85] partner repository with the dataset identifier PXD024034. RNA sequencing data are available upon request from the corresponding author.

## Abbreviations

CK: Control
LT: Low temperature
LTF: Low temperature flooding
DEPs: Differentially expressed proteins
DEGs: Differentially expressed genes
LC-MS: Liquid chromatograph- mass spectrometry
GO: Gene ontology
KEGG: Kyoto encyclopedia of genes and genomes
KOG: Klusters of eukaryotic orthologous groups
FC: Fold change
BP: Biological process
CC: Cellular component
MF: Molecular function
RH: Relative humidity
Rubisco: Ribulose bisphosphate carboxylase oxygenase
POD: Peroxidase
SOD: Superoxide dismutase
MDA: Malondialdehyde
PEPCK: Phosphoenolpyruvate carboxykinase
GA_3_: Gibberellin A_3_
ABA: Abscisic acid
FW: Fresh weight
HSD: Honst significant difference
