# Supplementary material for: Transcriptomic, proteomic, and physiological comparative analyses of flooding mitigation of the damage induced by low-temperature stress in direct seeded early indica rice at the seedling stage
Source: BMC Genomics. 2021 Mar 12;22:176. doi: 10.1186/s12864-021-07458-9 (PMC7952222; doi:10.1186/s12864-021-07458-9)
Supplement: Supplementary file 4 — Additional file 4 : Figure S1. Functional categories of the identified differentially expressed proteins (DEPs) between LT and CK. [file 12864_2021_7458_MOESM4_ESM.docx]

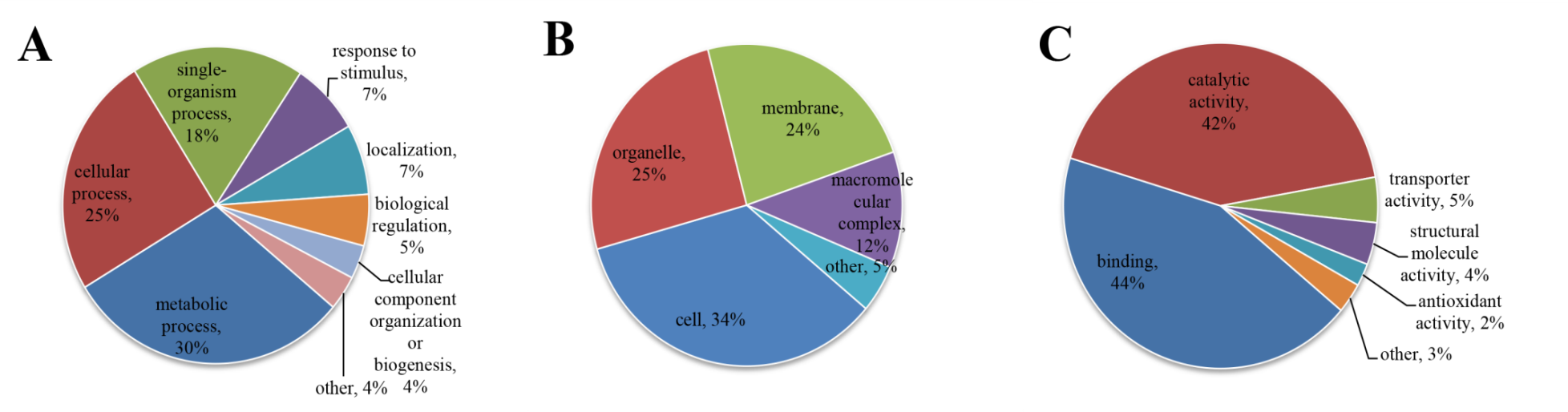


**Fig. S1** Functional categories of the identified differentially expressed proteins (DEPs) between LT and CK. A: Biological Process; B: Cellular Component; C: Molecular Function. LT: low temperature, LTF: low temperature flooding, CK: control.
