# Supplementary material for: Transcriptomic, proteomic, and physiological comparative analyses of flooding mitigation of the damage induced by low-temperature stress in direct seeded early indica rice at the seedling stage
Source: BMC Genomics. 2021 Mar 12;22:176. doi: 10.1186/s12864-021-07458-9 (PMC7952222; doi:10.1186/s12864-021-07458-9)
Supplement: Supplementary file 8 — Additional file 8 : Figure S5. DEPs protein–protein interaction analysis between LTF and CK. [file 12864_2021_7458_MOESM8_ESM.docx]

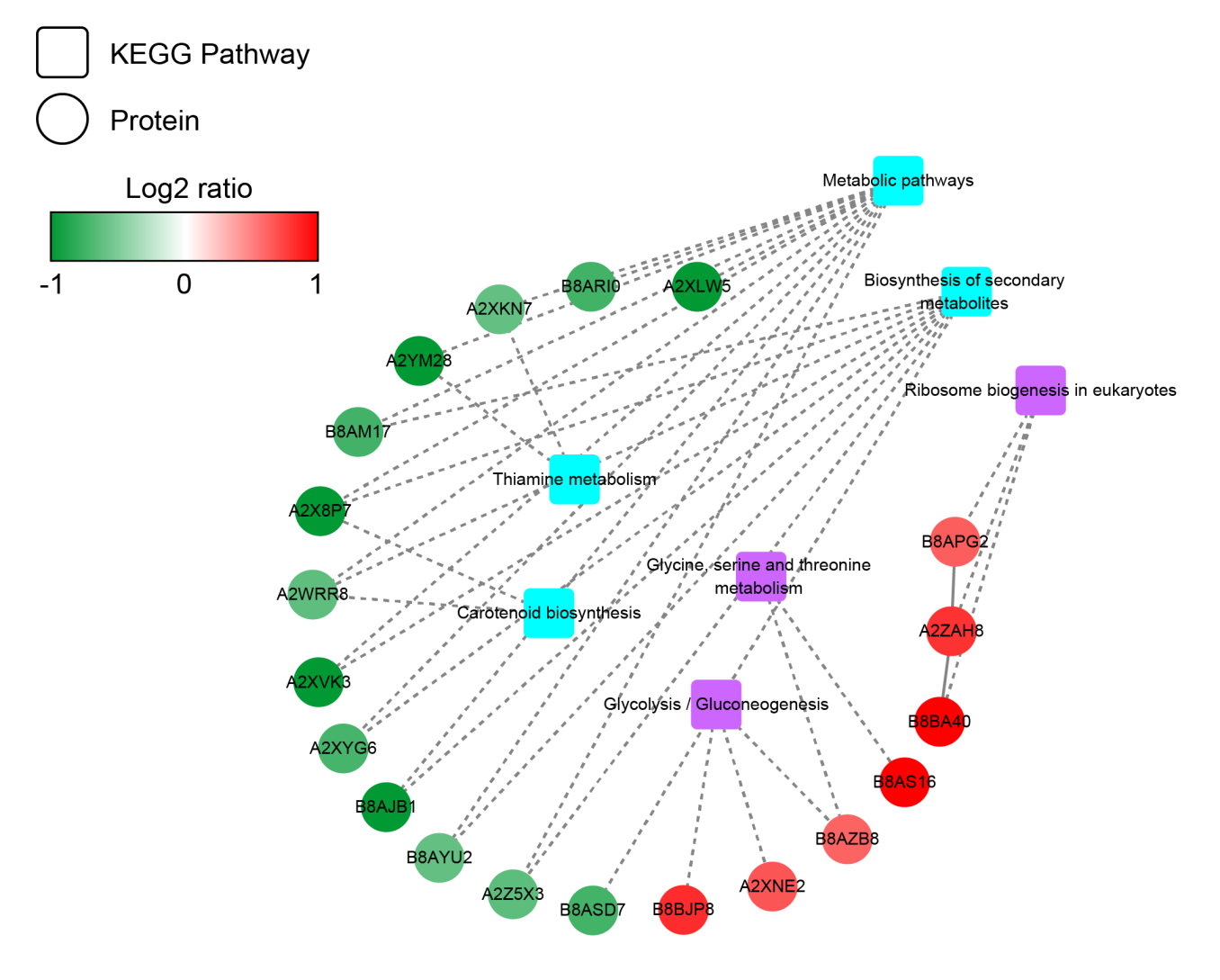


**Fig. S5** DEPs protein–protein interaction analysis between LTF and CK. LTF: low temperature flooding, CK: control.
