# Supplementary material for: Transcriptomic, proteomic, and physiological comparative analyses of flooding mitigation of the damage induced by low-temperature stress in direct seeded early indica rice at the seedling stage
Source: BMC Genomics. 2021 Mar 12;22:176. doi: 10.1186/s12864-021-07458-9 (PMC7952222; doi:10.1186/s12864-021-07458-9)
Supplement: Supplementary file 10 — Additional file 10 : Table S1. Effects of low-temperature and low temperature flooding on agronomic characters. [file 12864_2021_7458_MOESM10_ESM.docx]

**Table S1.** Effects of low-temperature and low temperature flooding on agronomic characters.

| Treatment | Seedling height /cm | Root number | Root length/cm | Fresh weigh  mg/100plant | Dry weigh  mg/100plant | T1  /cm | T2  /cm | T3 /cm |
| --- | --- | --- | --- | --- | --- | --- | --- | --- |
| LT | 18.9c | 10.7c | 5.3b | 186.0c | 27.2c | 2.4b | 9.3b | 7.1c |
| LTF | 19.9b | 11.0b | 5.5b | 212. 4b | 32.5b | 2.4b | 9.8b | 8.1b |
| CK | 28.4a | 14.1a | 7.9a | 253. 5a | 36.4a | 3.7a | 10.6a | 10.5a |

LT, low temperature; LTF, low temperature flooding; CK, control; T1, the first leaf from top; T2, the 2^nd^ leaf from top; T3, the 3^rd^ leaf from top. Different lowercase letters in the same column mean significantly different at 0.05 level.
