# Supplementary material for: Transcriptomic, proteomic, and physiological comparative analyses of flooding mitigation of the damage induced by low-temperature stress in direct seeded early indica rice at the seedling stage
Source: BMC Genomics. 2021 Mar 12;22:176. doi: 10.1186/s12864-021-07458-9 (PMC7952222; doi:10.1186/s12864-021-07458-9)
Supplement: Supplementary file 11 — Additional file 11 : Table S2. Primers used for qRT-PCR verification of differently expressed genes. [file 12864_2021_7458_MOESM11_ESM.docx]

**Table S2** Primers used for qRT-PCR verification of differently expressed genes.

| Gene | Forward primer | Reverse primer |
| --- | --- | --- |
| A2YM28 | CGTACGAGCTCTCCAAGGAC | ATGACGGTGGAGGTGAAGAG |
| A2XKN7 | GGATCCTGTGACCGCTATGT | TTCCTCCACTGTCCCGTAAC |
| A2WRR8 | GAAACCGTGTCTACCCTGGA | GAAGAAAGCGTCGAACCAAG |
| A2X8P7 | TGAAGTCCATGAGGCTTTCC | GCTACCAACTGCTCGTCCTC |
| B8AZB8 | TTCCCCCATTTGTGATTGTT | GGAAGCTTCAGCTCACCATC |
| B8BJP8 | ATCGAGGCCCTTATCCAGTT | GGCATTGGTGGCTTGTACTT |
| B8AS16 | TTGCCAAGAGTCATGGTCTG | CGCACAAAGAATTCCCACTT |
| A2XLW5 | AAGAGTTTGCCAGACGCAAT | CATGCTGCTTCATTCTCCAA |
| B8AYU2 | AAATTTCCCCAGGATGGTTC | GCCTGTGAAAGCTTGTCCTC |
| A2X822 | AGTGTGGCTTGACCAATTCC | GATGGGTGCAGTGTTGTCAC |
| B8ASV8 | AGCACCTACAAGGGGAAGGT | GAGTGCAAGCAAACTGGACA |
| A2YIT3 | GATGCTTGTGGTTCGGATCT | TGGACACCGGCTTCTTTTAC |
| A2XYC2 | GCACAGTTGAAGCACAGGAA | TCTGCGAGCACAACATAAGG |
| A2YMZ1 | ATCTTCATCCCGGAGTTCCT | GAGCTTGTTGTTGGGGAAGA |
| A2YCB9 | GACGATCCTGGTCATCGAGT | TCGTCCGTTCTTGATCTCCT |
| A2YHC5 | ACCATCTTCCAGCATCATCC | GGCTATGTAGATGCCGTTCC |
| A2YLE6 | CAGCACAAACAGCAGAGGAG | ATCGTAAGGCCCGTAGAGGT |
| B8B7M5 | CCTAGTGGGCGTTTTGGTAA | GTGGTCCCAACGGAGAAGTA |
| A2YP23 | GCTGTTATGGCGACTTGTGA | CACTCAGCTGGGGTCTTCTC |
| Rice 18s | ctacgtccctgccctttgtaca | acacttcaccggaccattcaa |
